# Increasing PACE of PACEMAKER Imaging!

**DOI:** 10.1186/1532-429X-18-S1-T6

**Published:** 2016-01-27

**Authors:** June A Yamrozik, Mark Doyle, Ronald B Williams, Geetha Rayarao, Diane V Thompson, Moneal Shah, Huma Samar, Robert W Biederman

**Affiliations:** 1 Cardiac MRI, Allegheny General Hospital, Pittsburgh, PA USA; 2 Cardiology, VA Medical Center, Loma, Linda, CA USA

## Background

Pacemaker/ AICD imaging is currently being done on patients in the MRI environment. A vigilant team consisting of the (Cardiologist, EP staff, and technologists) with close patient monitoring and supervision is making such an unmentionable procedure a success. However, is the diagnosis from imaging these patients adding valuable irrefutable information to merit such a risk?

## Methods

A total of 157 patients were imaged on a GE CV/i Excite Version 12, 1.5 T system (GE, Milwaukee, WI). 31 patients had an AICD, 13 patients had an AICD/Pacemaker, 6 patients had a single pacemaker lead, 14 REVO pacemakers and 93 patients had a complete pacemaker implantation. A specific criteria was followed for all the patients undergoing this procedure to scrutinize if the final diagnosis provided additional information in patient care. A checklist of three questions were gathered and answered after the final interpretation of the MRI.

1.Does the diagnosis change?

2.Does the MRI provide additional information to the existing diagnosis?

3.Does patient management change?

If yes was answered any of the above questions it was considered that the MRI scan was of value to patient diagnosis.

## Results

All patients completed the procedure with no adverse events and the pacemaker was interrogated after the procedure by EP Lab and reprogrammed under the direction of the Cardiologist. The average MRI scan time was 20 ± 55 min. A total of 157 patients imaged, 114 (73%) were neurology cases, 7musculoskeletal (4%) and 36(23%) were cardiac/vascular cases.

After reviewing the results from the 114 neurology cases 21 (18%) out of the 114 showed that the MRI not only provided additional information but changed the original diagnosis and in turn their course of medical treatment. 79 patients (69%) provided additional information to the diagnosis. Thus a total of 100 patients (88%) showed that the MRI scan was of value to the final diagnosis. 14(12%) out of the 114 patients imaged did not provide any further information but confirmed the original diagnosis. The 36 cardiac cases showed in 5 patients (14%) the MRI provided additional information to change the original diagnosis and also patient management. 28 (77%) showed that extra information was gathered. 3(8%) were unable to interpret due to artifact from AICD device. In essence, 92% of the Cardiac population benefited by having the MRI done. The 7 musculoskeletal demonstrated that 6(85%) provided additional information and 1(15%) changed patient management

## Conclusions

The use of PM/AICD imaging in MRI remains controversial but with a cautious cardiac team increased confidence in its use is found. Herein, we show that MRI procedures on carefully selected patients with pacemakers/AICD's are beneficial and substantially add valuable irrefutable information to patient diagnosis and management. We propose that not only are Pacemakers/AICD's no longer ***taboo***in the MRI environment but they can be markedly efficient with life-altering and life-saving consequences.

